# 2-Amino-5-cyano­pyridinium chloride

**DOI:** 10.1107/S1600536808020783

**Published:** 2008-07-12

**Authors:** Xiao-Chun Wen

**Affiliations:** a Ordered Matter Science Research Center, College of Chemistry and Chemical Engineering, Southeast University, Nanjing 210096, People’s Republic of China

## Abstract

In the crystal structure of the title compound, C_6_H_6_N_3_^+^·Cl^−^, cohesion is maintained by cation–anion N—H⋯Cl and cation–cation N—H⋯N hydrogen bonds, which link the ions into a three-dimensional network.

## Related literature

For the use of tetra­zole derivatives in coordination chemisty, see: Manzur *et al.* (2007 ▶); Ismayilov *et al.* (2007 ▶); Austria *et al.* (2007 ▶).
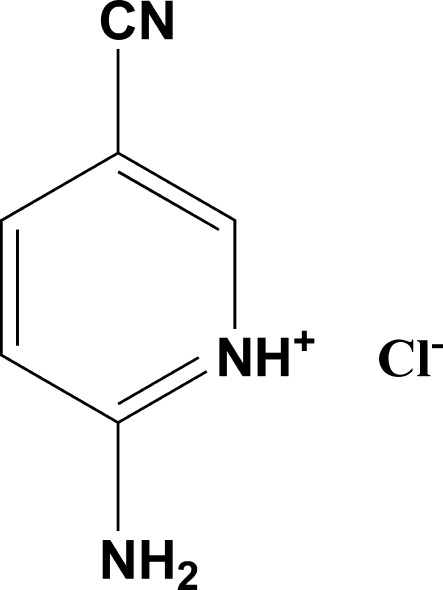


## Experimental

### 

#### Crystal data

C_6_H_6_N_3_^+^·Cl^−^
*M* *_r_* = 155.59
Monoclinic, 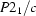

*a* = 4.0937 (8) Å
*b* = 11.856 (2) Å
*c* = 14.842 (3) Å
β = 94.95 (3)°
*V* = 717.7 (2) Å^3^
*Z* = 4
Mo *K*α radiation
μ = 0.45 mm^−1^
*T* = 298 (2) K
0.18 × 0.15 × 0.15 mm

#### Data collection

Rigaku Mercury2 diffractometer
Absorption correction: multi-scan (*CrystalClear*; Rigaku, 2005 ▶) *T*_min_ = 0.922, *T*_max_ = 0.935
7307 measured reflections
1652 independent reflections
1252 reflections with *I* > 2σ(*I*)
*R*_int_ = 0.044

#### Refinement

*R*[*F* ^2^ > 2σ(*F* ^2^)] = 0.045
*wR*(*F* ^2^) = 0.102
*S* = 1.06
1652 reflections
91 parameters
H-atom parameters constrained
Δρ_max_ = 0.21 e Å^−3^
Δρ_min_ = −0.23 e Å^−3^

### 

Data collection: *CrystalClear* (Rigaku, 2005 ▶); cell refinement: *CrystalClear*; data reduction: *CrystalClear*; program(s) used to solve structure: *SHELXS97* (Sheldrick, 2008 ▶); program(s) used to refine structure: *SHELXL97* (Sheldrick, 2008 ▶); molecular graphics: *SHELXTL/PC* (Sheldrick, 2008 ▶); software used to prepare material for publication: *SHELXTL/PC*.

## Supplementary Material

Crystal structure: contains datablocks I, global. DOI: 10.1107/S1600536808020783/rz2232sup1.cif

Structure factors: contains datablocks I. DOI: 10.1107/S1600536808020783/rz2232Isup2.hkl

Additional supplementary materials:  crystallographic information; 3D view; checkCIF report

## Figures and Tables

**Table 1:** Hydrogen-bond geometry (Å, °)

*D*—H⋯*A*	*D*—H	H⋯*A*	*D*⋯*A*	*D*—H⋯*A*
N2—H2*A*⋯Cl1	0.86	2.29	3.0818 (18)	153
N3—H3*A*⋯Cl1	0.86	2.65	3.363 (2)	141
N3—H3*A*⋯N1^i^	0.86	2.53	3.046 (3)	120
N3—H3*B*⋯Cl1^ii^	0.86	2.37	3.216 (2)	167

Symmetry codes: (i) 
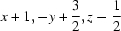
; (ii) 
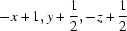
.

**Fig. 1.**
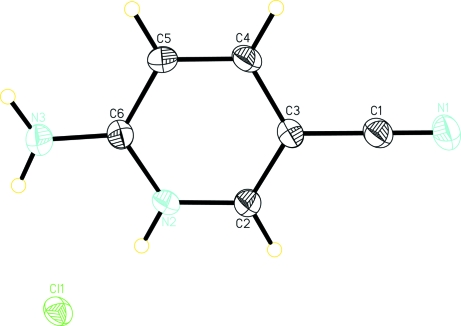
A view of the title compound with the atom-numbering scheme. Displacement ellipsoids were drawn at the 30% probability level.

**Fig. 2.**
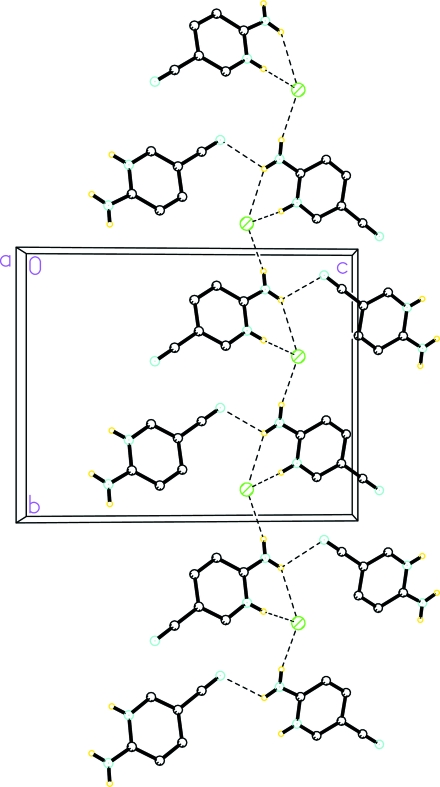
Partial crystal packing of the title compound viewed along the a axis showing H bonding pattern as dashed lines. Hydrogen atoms not involved in hydrogen bonding are omitted for clarity.

## supplementary crystallographic information

### Comment

In the past five years, we have focused on the chemistry of amine derivatives because of their multiple coordination modes as ligands to metal ions and for the construction of novel metal-organic frameworks (Manzur *et al.* 2007; Ismayilov *et al.* 2007; Austria *et al.* 2007). Herein the crystal structure of the title compound, 6-aminonicotinonitrile-1-ium chloride, is reported.

In the title compound (Fig.1), the N2 atom of the pyridine ring is protonated. The nitrile group and the pyridine ring are nearly coplanar, as indicated by the dihedral angle of 86.71 (14)° formed by the C≡N vector with the normal to the pyridine plane. Crystal cohesion is enforced by cation-anion N—H···Cl and cation-cation N—H···N hydrogen bonds (Table 1,  Fig. 2) linking molecules into a three-dimensional network.

### Experimental

6-Aminonicotinonitrile-1-ium chloride (3 mmol) was dissolved in ethanol (20 ml) and evaporated in the air affording colourless block-shaped crystals suitable for X-ray analysis.

### Refinement

All H atoms were fixed geometrically and treated as riding, with C–H = 0.93 Å, N–H =0.86 Å, and with *U*_iso_(H) =1.2Ueq(C, N).

### Crystal data

**Table:**

C_6_H_6_N_3_^+^·Cl^–^	*F*_000_ = 320
*M**_r_* = 155.59	*D*_x_ = 1.440 Mg m^−^^3^
Monoclinic, *P*2_1_/*c*	Mo *K*α radiation λ = 0.71073 Å
Hall symbol: -P 2ybc	Cell parameters from 1250 reflections
*a* = 4.0937 (8) Å	θ = 2.3–24.4º
*b* = 11.856 (2) Å	µ = 0.45 mm^−^^1^
*c* = 14.842 (3) Å	*T* = 298 (2) K
β = 94.95 (3)º	Block, colourless
*V* = 717.7 (2) Å^3^	0.18 × 0.15 × 0.15 mm
*Z* = 4	

### Data collection

**Table:**

Rigaku Mercury2 diffractometer	1652 independent reflections
Radiation source: fine-focus sealed tube	1252 reflections with *I* > 2σ(*I*)
Monochromator: graphite	*R*_int_ = 0.044
Detector resolution: 13.6612 pixels mm^-1^	θ_max_ = 27.5º
*T* = 298(2) K	θ_min_ = 3.3º
ω scans	*h* = −5→5
Absorption correction: multi-scan(CrystalClear; Rigaku, 2005)	*k* = −15→15
*T*_min_ = 0.922, *T*_max_ = 0.935	*l* = −19→19
7307 measured reflections	

### Refinement

**Table:**

Refinement on *F*^2^	Secondary atom site location: difference Fourier map
Least-squares matrix: full	Hydrogen site location: inferred from neighbouring sites
*R*[*F*^2^ > 2σ(*F*^2^)] = 0.045	H-atom parameters constrained
*wR*(*F*^2^) = 0.102	*w* = 1/[σ^2^(*F*_o_^2^) + (0.041*P*)^2^ + 0.2213*P*] where *P* = (*F*_o_^2^ + 2*F*_c_^2^)/3
*S* = 1.06	(Δ/σ)_max_ < 0.001
1652 reflections	Δρ_max_ = 0.21 e Å^−^^3^
91 parameters	Δρ_min_ = −0.23 e Å^−^^3^
Primary atom site location: structure-invariant direct methods	Extinction correction: none

### Special details

**Table:**

Geometry. All e.s.d.'s (except the e.s.d. in the dihedral angle between two l.s. planes) are estimated using the full covariance matrix. The cell e.s.d.'s are taken into account individually in the estimation of e.s.d.'s in distances, angles and torsion angles; correlations between e.s.d.'s in cell parameters are only used when they are defined by crystal symmetry. An approximate (isotropic) treatment of cell e.s.d.'s is used for estimating e.s.d.'s involving l.s. planes.
Refinement. Refinement of *F*^2^ against ALL reflections. The weighted *R*-factor *wR* and goodness of fit *S* are based on *F*^2^, conventional *R*-factors *R* are based on *F*, with *F* set to zero for negative *F*^2^. The threshold expression of *F*^2^ > σ(*F*^2^) is used only for calculating *R*-factors(gt) *etc*. and is not relevant to the choice of reflections for refinement. *R*-factors based on *F*^2^ are statistically about twice as large as those based on *F*, and *R*- factors based on ALL data will be even larger.

### Fractional atomic coordinates and isotropic or equivalent isotropic displacement parameters (Å^2^)

**Table:**

	*x*	*y*	*z*	*U*_iso_*/*U*_eq_	
Cl1	0.44244 (14)	0.60704 (5)	0.17252 (4)	0.0459 (2)	
N2	0.0652 (4)	0.69979 (14)	0.32749 (11)	0.0377 (4)	
H2A	0.1041	0.6650	0.2787	0.045*	
C2	−0.0884 (5)	0.64335 (18)	0.39019 (14)	0.0385 (5)	
H2B	−0.1506	0.5687	0.3801	0.046*	
N3	0.3183 (5)	0.85595 (16)	0.27271 (13)	0.0507 (5)	
H3A	0.3567	0.8175	0.2256	0.061*	
H3B	0.3816	0.9250	0.2777	0.061*	
C3	−0.1527 (5)	0.69550 (17)	0.46837 (13)	0.0352 (5)	
C1	−0.3034 (6)	0.63422 (18)	0.53757 (15)	0.0442 (5)	
C6	0.1619 (5)	0.80885 (17)	0.33739 (13)	0.0355 (5)	
C4	−0.0595 (5)	0.81008 (17)	0.48174 (14)	0.0398 (5)	
H4A	−0.1029	0.8471	0.5346	0.048*	
N1	−0.4184 (6)	0.58515 (18)	0.59282 (14)	0.0626 (6)	
C5	0.0921 (5)	0.86506 (17)	0.41756 (15)	0.0414 (5)	
H5A	0.1510	0.9403	0.4261	0.050*	

### Atomic displacement parameters (Å^2^)

**Table:**

	*U*^11^	*U*^22^	*U*^33^	*U*^12^	*U*^13^	*U*^23^
Cl1	0.0522 (3)	0.0472 (3)	0.0398 (3)	0.0063 (3)	0.0124 (2)	−0.0055 (2)
N2	0.0471 (10)	0.0364 (9)	0.0305 (9)	−0.0004 (8)	0.0091 (7)	−0.0073 (7)
C2	0.0442 (12)	0.0344 (11)	0.0376 (11)	−0.0020 (9)	0.0082 (9)	−0.0007 (9)
N3	0.0672 (13)	0.0442 (11)	0.0435 (11)	−0.0080 (10)	0.0207 (10)	−0.0001 (9)
C3	0.0370 (11)	0.0378 (11)	0.0313 (10)	0.0033 (9)	0.0063 (8)	0.0016 (8)
C1	0.0523 (13)	0.0414 (12)	0.0400 (12)	0.0027 (10)	0.0101 (10)	−0.0022 (10)
C6	0.0370 (11)	0.0366 (11)	0.0332 (10)	0.0022 (9)	0.0054 (9)	0.0028 (8)
C4	0.0486 (12)	0.0384 (11)	0.0334 (11)	0.0036 (10)	0.0104 (9)	−0.0066 (9)
N1	0.0862 (16)	0.0547 (13)	0.0514 (12)	−0.0085 (11)	0.0316 (12)	0.0009 (10)
C5	0.0530 (13)	0.0306 (11)	0.0419 (12)	−0.0015 (9)	0.0106 (10)	−0.0060 (9)

### Geometric parameters (Å, °)

**Table:**

N2—C2	1.345 (2)	C3—C4	1.420 (3)
N2—C6	1.356 (3)	C3—C1	1.440 (3)
N2—H2A	0.8600	C1—N1	1.140 (3)
C2—C3	1.360 (3)	C6—C5	1.414 (3)
C2—H2B	0.9300	C4—C5	1.349 (3)
N3—C6	1.323 (3)	C4—H4A	0.9300
N3—H3A	0.8600	C5—H5A	0.9300
N3—H3B	0.8600		
			
C2—N2—C6	123.26 (17)	C4—C3—C1	120.62 (18)
C2—N2—H2A	118.4	N1—C1—C3	179.0 (3)
C6—N2—H2A	118.4	N3—C6—N2	118.60 (18)
N2—C2—C3	120.02 (19)	N3—C6—C5	123.9 (2)
N2—C2—H2B	120.0	N2—C6—C5	117.53 (18)
C3—C2—H2B	120.0	C5—C4—C3	119.85 (18)
C6—N3—H3A	120.0	C5—C4—H4A	120.1
C6—N3—H3B	120.0	C3—C4—H4A	120.1
H3A—N3—H3B	120.0	C4—C5—C6	120.34 (19)
C2—C3—C4	118.98 (18)	C4—C5—H5A	119.8
C2—C3—C1	120.37 (19)	C6—C5—H5A	119.8
			
C6—N2—C2—C3	−0.3 (3)	C2—C3—C4—C5	−0.4 (3)
N2—C2—C3—C4	0.9 (3)	C1—C3—C4—C5	177.6 (2)
N2—C2—C3—C1	−177.1 (2)	C3—C4—C5—C6	−0.7 (3)
C2—N2—C6—N3	178.4 (2)	N3—C6—C5—C4	−177.9 (2)
C2—N2—C6—C5	−0.8 (3)	N2—C6—C5—C4	1.2 (3)

### Hydrogen-bond geometry (Å, °)

**Table:**

*D*—H···*A*	*D*—H	H···*A*	*D*···*A*	*D*—H···*A*
N2—H2A···Cl1	0.86	2.29	3.0818 (18)	153
N3—H3A···Cl1	0.86	2.65	3.363 (2)	141
N3—H3A···N1^i^	0.86	2.53	3.046 (3)	120
N3—H3B···Cl1^ii^	0.86	2.37	3.216 (2)	167

Symmetry codes: (i) *x*+1, −*y*+3/2, *z*−1/2; (ii) −*x*+1, *y*+1/2, −*z*+1/2.
